# Crystal structure of the lytic CHAP_K_ domain of the endolysin LysK from *Staphylococcus aureus* bacteriophage K

**DOI:** 10.1186/1743-422X-11-133

**Published:** 2014-07-26

**Authors:** Marta Sanz-Gaitero, Ruth Keary, Carmela Garcia-Doval, Aidan Coffey, Mark J van Raaij

**Affiliations:** 1 Departamento de Estructura de Macromoleculas, Centro Nacional de Biotecnologia (CNB–CSIC), Calle Darwin 3, E-28049 Madrid, Spain; 2 Department of Biological Sciences, Cork Institute of Technology, Bishopstown, Cork, Ireland; 3 Current address: Department of Biochemistry, University of Zurich, Zurich, Switzerland

**Keywords:** Bacteriophage, Calcium, Crystallography, Endolysin, Peptidoglycan, Protease, *Staphylococcus*, Zinc

## Abstract

**Background:**

Bacteriophages encode endolysins to lyse their host cell and allow escape of their progeny. Endolysins are also active against Gram-positive bacteria when applied from the outside and are thus attractive anti-bacterial agents. LysK, an endolysin from staphylococcal phage K, contains an N-terminal cysteine-histidine dependent amido-hydrolase/peptidase domain (CHAP_K_), a central amidase domain and a C-terminal SH3b cell wall-binding domain. CHAP_K_ cleaves bacterial peptidoglycan between the tetra-peptide stem and the penta-glycine bridge.

**Methods:**

The CHAP_K_ domain of LysK was crystallized and high-resolution diffraction data was collected both from a native protein crystal and a methylmercury chloride derivatized crystal. The anomalous signal contained in the derivative data allowed the location of heavy atom sites and phase determination. The resulting structures were completed, refined and analyzed. The presence of calcium and zinc ions in the structure was confirmed by X-ray fluorescence emission spectroscopy. Zymogram analysis was performed on the enzyme and selected site-directed mutants.

**Results:**

The structure of CHAP_K_ revealed a papain-like topology with a hydrophobic cleft, where the catalytic triad is located. Ordered buffer molecules present in this groove may mimic the peptidoglycan substrate. When compared to previously solved CHAP domains, CHAP_K_ contains an additional lobe in its N-terminal domain, with a structural calcium ion, coordinated by residues Asp45, Asp47, Tyr49, His51 and Asp56. The presence of a zinc ion in the active site was also apparent, coordinated by the catalytic residue Cys54 and a possible substrate analogue. Site-directed mutagenesis was used to demonstrate that residues involved in calcium binding and of the proposed active site were important for enzyme activity.

**Conclusions:**

The high-resolution structure of the CHAP_K_ domain of LysK was determined, suggesting the location of the active site, the substrate-binding groove and revealing the presence of a structurally important calcium ion. A zinc ion was found more loosely bound. Based on the structure, we propose a possible reaction mechanism. Future studies will be aimed at co-crystallizing CHAP_K_ with substrate analogues and elucidating its role in the complete LysK protein. This, in turn, may lead to the design of site-directed mutants with altered activity or substrate specificity.

## Abstract (es)

**Spanish abstract:**

**Introducción**

Los bacteriófagos codifican endolisinas para lisar sus bacterias hospedadoras y permitir la liberación de su progenie. Las endolisinas también son activas contra bacterias Gram positivas cuando se aplican desde el exterior, y por lo tanto, son consideradas agentes antibacterianos atractivos. LysK, una endolisina del fago K que infecta estafilococos, contiene un dominio N-terminal amidohidrolasa/peptidasa dependiente de cisteína e histidina (CHAP_K_), un dominio amidasa central y un dominio C-terminal SH3b de unión a la pared bacteriana. CHAP_K_ corta el peptidoglicano bacteriano entre el tetrapéptido y los puentes pentaglicina.

**Métodos**

El dominio CHAP_K_ de LysK fue cristalizado y se obtuvieron datos de difracción a alta resolución tanto de un cristal de proteína nativo como de un cristal derivado con cloruro de metilmercurio. La señal anómala presente en los datos derivados permitió la localización de la posición de los átomos pesados y la determinación de la fase. Las estructuras resultantes se completaron, refinaron y analizaron. La presencia de iones de calcio y zinc en la estructura fue confirmada por espectroscopía de emisión de fluorescencia de rayos X. Se llevaron a cabo análisis de zimograma sobre la enzima nativa y sobre mutantes puntuales seleccionados.

**Resultados**

La estructura de CHAP_K_ reveló una topología tipo papaína con un bolsillo hidrofóbico donde se localiza la tríada catalítica. Moléculas de tampón ordenadas presentes en este hueco pueden mimetizar el substrato de peptidoglicano. Cuando se compara con dominios CHAP resueltos previamente, CHAP_K_ contiene un lóbulo adicional en su dominio N-terminal, con un ión de calcio estructural, coordinado por los residuos Asp56, Asp45, Asp47. También se observa la presencia de un ión de zinc en el centro activo, coordinado con el residuo catalítico Cys54 y un posible análogo del substrato. Se usó mutagénesis dirigida para demostrar que los residuos involucrados en la unión a calcio y los presentes en el centro activo propuesto eran importantes para la actividad enzimática.

**Conclusiones**

Se determinó la estructura del dominio CHAP_K_ de LysK a alta resolución, sugiriendo la localización del centro activo, del bolsillo de unión al sustrato y revelando la presencia de un ión de calcio estructuralmente importante. Se encontró un ión de zinc unido más débilmente. Basándonos en la estructura, proponemos un posible mecanismo de reacción. Futuros estudios tendrán por objeto la cristalización de CHAP_K_ con análogos del sustrato y la elucidación de su papel en la proteína LysK completa. Esto, a su vez, podría conducir al diseño de mutantes puntuales con una actividad o especificidad de sustrato modificada.

## Background

Bacteriophage K is a virulent phage that infects a wide range of staphylococci. It belongs to the *Myoviridae* family of the *Caudovirales* order, with a genome of 148,317 bp [1-3]. To allow its progeny to escape from the host cell (“lysis from within”), it encodes the endolysin LysK, a peptidoglycan hydrolase [4]. When applied exogenously to the pathogen, LysK causes “lysis from without” or exolysis [5]. Gram-positive endolysins are highly specific [4], and no bacterial variants resistant to their phage endolysins have been found despite the use of mutagenesis strategies to promote the chance of resistance development [6]. LysK kills a wide range of staphylococci, including multi-drug-resistant *Staphylococcus aureus* (MRSA) [7].

LysK contains three domains: an N-terminal cysteine-histidine dependent amido-hydrolase/peptidase (CHAP) domain, a central amidase domain and a C-terminal SH3b cell wall-binding domain. The LysK amidase domain cleaves peptidoglycan between N-acetylmuramic acid and L-alanine of the stem peptide, while the CHAP domain hydrolyzes it between the D-alanine of the tetra-peptide stem and the first glycine of the penta-glycine cross-bridge [8]. A truncated enzyme called CHAP_K_, containing only the first 165 amino acids of LysK corresponding to the CHAP domain, also showed exolytic activity [9]. CHAP_K_ is able to lyse several staphyloccocal species, independently from their origin, their antibiotic resistance profile and their ability to produce exopolysaccharides (associated with biofilm formation) [10,11]. It is also effective against other related genera, such as *Micrococcus* or *Streptococcus*[7].

In order to understand the reaction mechanism and perhaps improve or alter the activity, we set out to solve the structure of CHAP_K_. The CHAP_K_ domain was expressed in *Escherichia coli*, purified and crystallized. Although the crystallization procedure was not very reproducible and crystals grew as inter-grown plates, a high-resolution dataset could be collected from one of them, plus a dataset from a methylmercury chloride derivative of sufficient quality for structure solution by single-wavelength anomalous dispersion [12]. This structure was refined against both the native and the derivative dataset. Here we present the high-resolution structure of the CHAP_K_ domain solved by X-ray crystallography.

## Results and discussion

### Overall structure

The final models of the CHAP_K_ enzyme contain amino acids 2–165 for each of the four protein molecules present in the crystallographic asymmetric units, with good crystallographic statistics and reasonable protein geometry (Table 1). The models also contain metal ions, waters and other solvent molecules. For the native structure, a calcium ion, a zinc ion and a 2-(N-morpholino)ethanesulfonic acid (MES) molecule have been modelled associated with each of the protein chains, as discussed below. Other ordered solvent molecules have also been modelled in the asymmetric unit and consist of one glycerol molecule, four putative sodium ions and 741 water molecules. For the derivative structure, a calcium ion and a 2-[4-(2-hydroxyethyl)piperazin-1-yl] ethanesulfonic acid (HEPES) molecule have been modelled associated with each of the protein chains, while Cys54 is modelled as methylmercury-cysteine. In this case, ordered solvent molecules modelled in the unit cell include two glycerol molecules, ten additional putative methylmercury ions, two putative chloride ions and 770 waters. Despite the lower nominal resolution of the native dataset when compared with the derivative (1.8 vs. 1.7 Å), the general structural analyses described below are done using the structure refined against the native dataset, as that dataset is more complete (97.2 vs. 64.7%), contains more measured reflections (62028 vs. 48498) [10], and better maps with less non-interpretable noise peaks were obtained.

**Table 1:** **Refinement and validation statistics for the CHAP**_**K **_**structure**

	**Native**	**Derivative**
PDB code	4CSH	4CT3
Space group	*P*1	*P*1
Cell edges (a, b, c, Å)	39.2, 61.5, 73.2	39.0, 61.5, 72.8
Cell angles (α, β, γ, º)	91.5, 98.7, 90.1	91.8, 98.7, 90.0
Resolution range used (Å)	32.9-1.79 (1.88-1.79)^a^	61.5-1.69 (1.78-1.69)
Multiplicity	2.0 (1.9)	3.4 (3.2)
Completeness	97.2 (94.3)	64.7 (10.4)
Mean <I/sigma(I)>	6.3 (2.9)	11.8 (1.8)
R_sym_ (%)^b^	9.1 (25.1)	6.0 (62.0)
Number of reflections used	59686 (8628)	46067 (1349)
Number of reflections used for R-free	2338 (112)	2431 (62)
R-factor^c^	0.175 (0.233)	0.181 (0.278)
R-free	0.201 (0.282)	0.224 (0.295)
Number of atoms (protein/water/other)	5286/741/66	5259/770/104
Average B-value/Wilson B-value (Å^2^)	18.4/14.5	22.2/14.9
Ramachandran statistics^d^ (%)	97.7/100.0	98.2/100.0
R.m.s. deviations^e^ (bonds, Å/angles, °)	0.015/1.49	0.012/1.37

^a^Values in parentheses are for the highest resolution bin, where applicable.

^b^R_sym_=Σ_h_Σ_i_|I_hi_-<I_h_>|/Σ_h_Σ_i_|I_hi_|, where I_hi_ is the intensity of the *i*th measurement of the same reflection and <I_h_> is the mean observed intensity for that reflection.

^c^R=Σ||*Fobs(hkl)*|-|*Fcalc(hkl)*||/Σ|*Fobs(hkl)*|.

^d^Determined with MOLPROBITY. The percentages are indicated of residues in favoured and allowed regions of the Ramachandran plot, respectively.

^e^Provided by REFMAC.

The four CHAP_K_ monomers do not form extensive inter-monomer interfaces in the crystal, suggesting that in solution the protein is monomeric. When the four crystallographically independent monomers are compared with each other, it is observed that they are very similar. While in part this is due to the use of local non-crystallographic symmetry restraints in the refinement, the fact that including these restraints significantly improved correspondence of the model to the data supports the similarity of the four crystallographically independent protein chains. Chains A and B on one hand, and chains C and D on the other, can be most reliably superposed, with root mean square differences (r.m.s.d.) between C-alpha atoms of 0.07 and 0.05 Å, respectively. The r.m.s.d. between chains A or B on one hand and chains C or D on the other are 0.23-0.26 Å. The largest structural differences are concentrated in residues 29–39 and 136–143, part of surface loops that interact with each other. These differences between the monomers are likely caused by interaction with neighbouring monomers in the crystal, i.e. different crystal contacts. The loop consisting of residues 136 to 143 is right next to a putative substrate-binding groove, so it may be somewhat more flexible to allow access of the substrate and release of the cleavage products.

**Figure 1:**
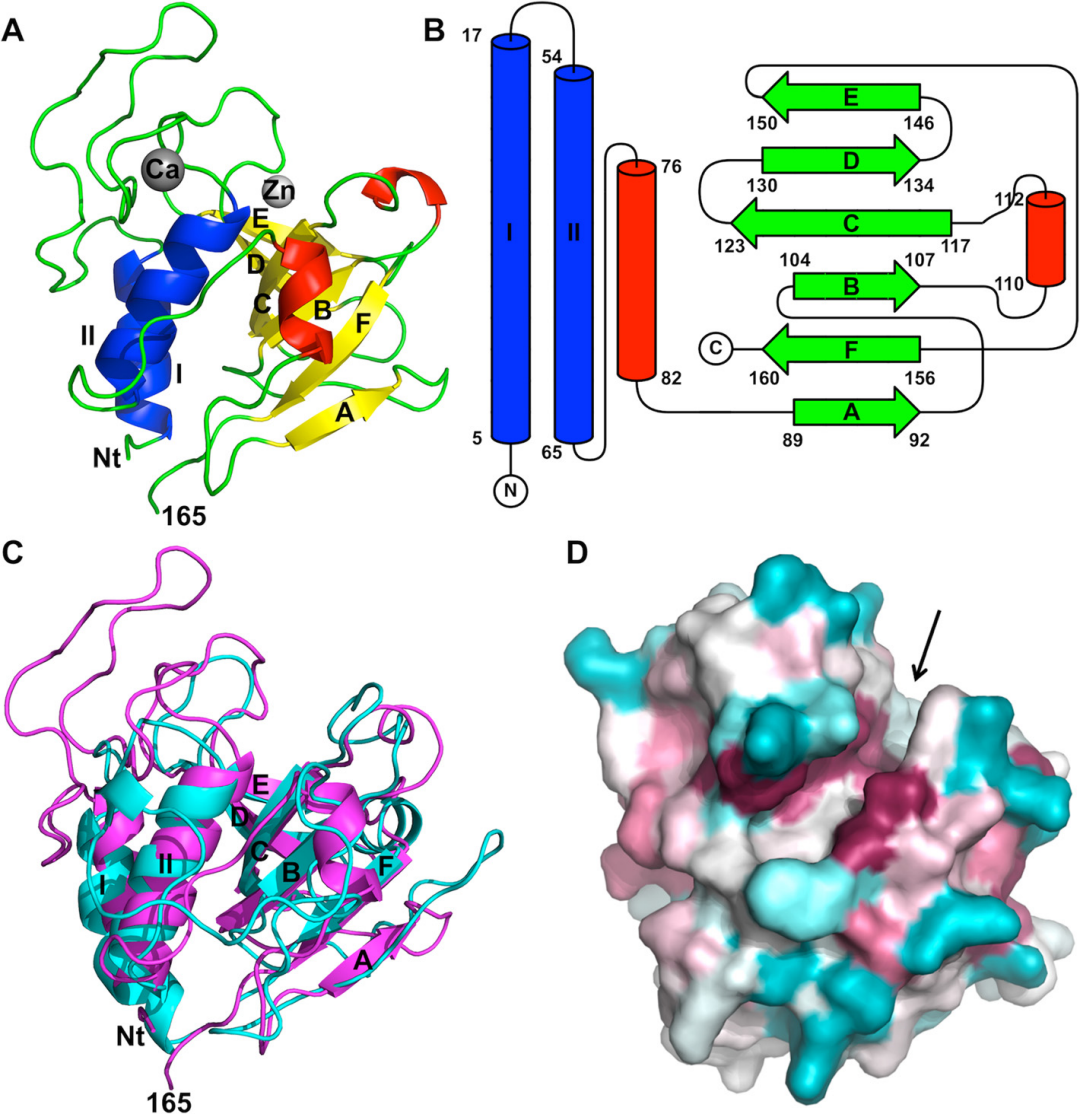
**Crystal structure of the N-terminal cysteine-histidine dependent amido-hydrolase/peptidase domain (CHAP**_**K**_**) of the endolysin LysK from staphylococcal bacteriophage K. (A)** Overall structure. Beta-strands are shown in green, alpha-helices in blue and 3_10_-helices in red. The calcium ion is shown in grey, the zinc ion in white. The N-terminal end (Nt), residue 165, the alpha-helices and the beta-strands are labelled. **(B)**. Topology diagram. The same labelling is used as in panel **A**. **(C)**. Superposition of CHAP_K_ (magenta) onto structure onto the CHAP domain of the streptococcal phage endolysin PlyC (PDB entry 4 F88; cyan). **(D)**. Space-filling representation with conserved residues in almost the same orientation as panel **A**, but slightly tilted forward to better illustrate the hydrophobic groove, which is indicated with an arrow. The colour coding goes from blue for less conserved residues, via white, to purple for the most conserved residues.

The CHAP_K_ protein consists of a single globular domain that contains two alpha-helices, two 3_10_-helices and six beta-strands (Figure 1A and B). The amino-terminal part of the protein consists of the two alpha-helices (I and II) interconnected by a long loop. This long loop borders a groove in the protein, at the bottom of which the catalytic site is located (see below). Another loop, containing a 3_10_-helix, connects this amino-terminal part of the protein to a six-stranded beta-sheet that forms the carboxy-terminal part. The six beta-strands are arranged in an anti-parallel beta-sheet in the topology AFBCDE (Figure 1B). The structure of CHAP_K_ had previously been predicted by *in silico* modelling [13]. The six-stranded beta-sheet was predicted well, but the amino-terminal alpha-helices were incorrectly placed and the calcium-binding loop between them was not present in the model. The main chain atoms of the catalytic site residues were within 2 Å of their predicted positions.

When the structure is analyzed, it is clear that CHAP_K_ belongs to the cysteine protease CA peptidase clan Pfam: CL0125; http://pfam.xfam.org/; Ref. [14], with a papain-like fold. CHAP_K_ is a member of the CHAP family of this clan (Pfam: PF05257), as expected from sequence homology. A structural similarity search revealed that the most similar structure is the CHAP domain of the streptococcal phage endolysin PlyC (PDB entry 4F88) [15], with a root mean square difference (r.m.s.d.) of 2.5 Å when the backbone atoms of 124 residues are superposed onto CHAP_K_ (Z-score 11.4). The next most similar structure is the C-terminal endopeptidase domain of the NlpC/P60 family cell-wall remodelling protein *Bacillus cereus* PDB code 3H41; Ref. [16], with an r.m.s.d. of 2.8 Å when the backbone atoms of 114 residues are superposed (Z-score 10.2). When the PDB database is searched for sequence-similar structures, the first hit is the CHAP domain from *Staphylococcus saprophyticus* CHAP domain protein (PDB entry 2K3A) [17], with a sequence identity of 28% over a stretch of 94 residues. However, this structure cannot be superimposed as well as those previously mentioned (r.m.s.d. of 3.4 Å when backbone atoms of 101 residues are superposed, Z-score 6.3) and our attempts to solve the CHAP_K_ structure by molecular replacement using this model were unsuccessful. This lower similarity may be due to the fact that this structure was determined by NMR spectroscopy rather than crystallography. Superposition of the CHAP_K_ structure onto the CHAP domain of the streptococcal phage endolysin PlyC (PDB entry 4F88) is shown in Figure 1C. The two alpha-helices and six beta-strands of CHAP_K_ superpose quite well with the backbone of the homologous structures, but the loops, including the 3_10_-helices, are very different.

**Figure 2:**
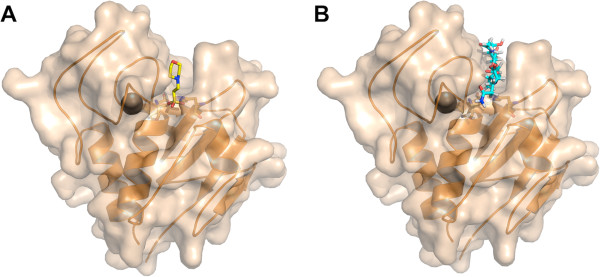
**MES buffer molecule bound to the CHAP**_**K **_**enzyme putative substrate binding site.** The CHAP_K_ protein is shown in transparent surface and secondary structure cartoon representation; the calcium ion is also shown.

The globular CHAP_K_ protein has a relatively long and deep hydrophobic groove. When sequence conservation is mapped onto the surface, one notices that several residues lining the groove are highly conserved (Figure 1D; the sequence alignment underlying this figure is in Additional file 1: Table S1). In the native structure, a MES molecule is located in this groove (Figure 2, PDB entry 4CSH), while in the derivative structure a HEPES molecule is present (PDB entry 4CT3). These molecules may well be mimicking the natural peptidoglycan substrate of the protein. Residues in the groove that might contact the peptidoglycan substrate are: Phe36, Asp47, Tyr49, Tyr50, Gln53 and Cys54 from the loop between helices 1 and 2; Asp56 and Thr59 from helix 2; Arg71, Trp73 and Asn75 from the loop between helix 2 and beta-strand A; Trp115 and His117 from the BC-loop and Asn136 and Trp137 from the DE-loop.

### Bound metal ions

**Figure 3:**
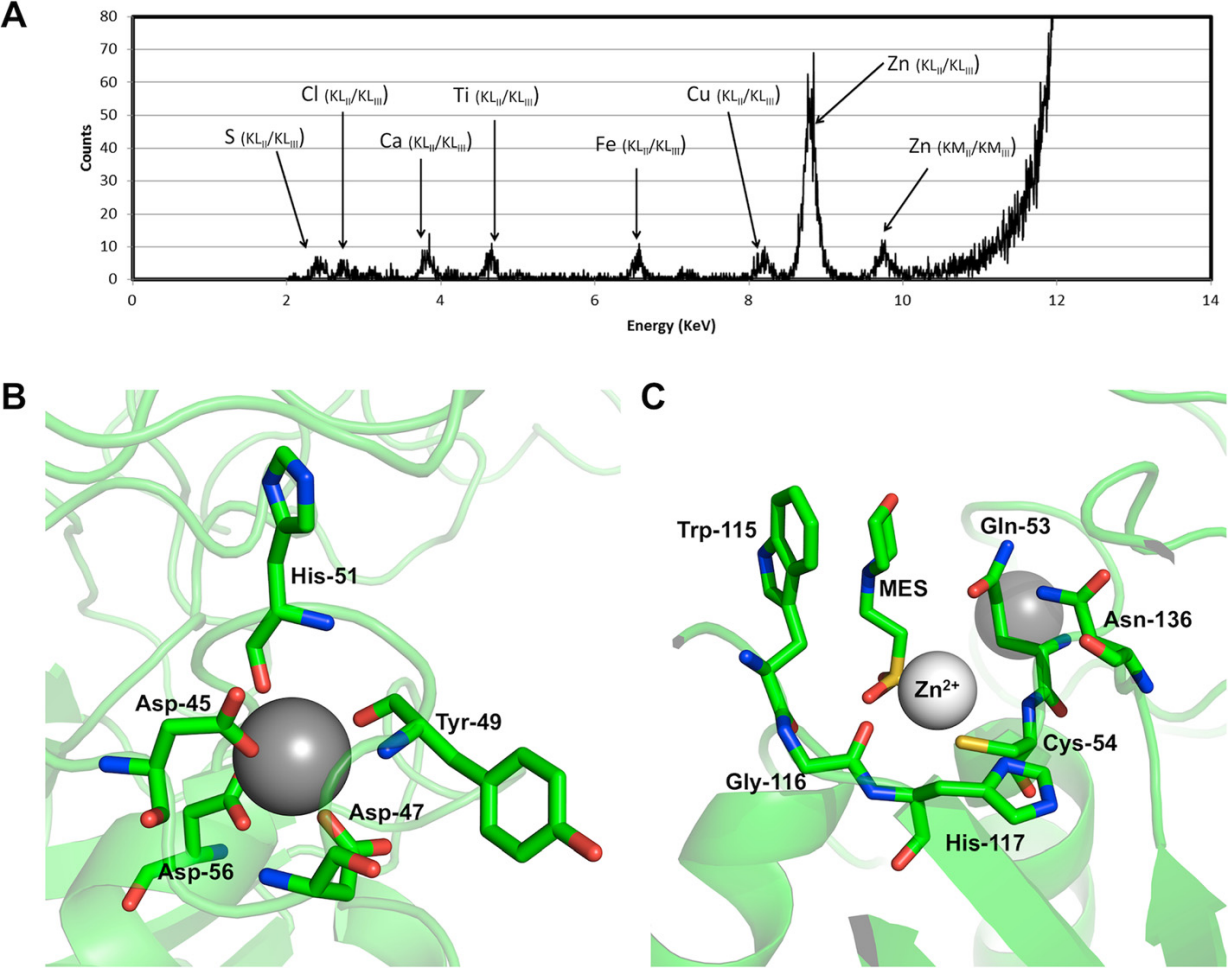
**Presence of metal ions in the CHAP**_**K **_**crystal structure. A**. X-ray fluorescence emission spectrum collected from a CHAP_K_ crystal irradiated with monochromatic synchrotron radiation (12.7 KeV). **B**. Detail of the calcium ion coordination. Coordinating atoms are one Oδ atom of each of Asp45 and Asp47 residues, both Oδ atoms of Asp56, the main chain oxygen atoms of Tyr49 and His51 and an ordered water molecule (behind the calcium ion in this view). **C**. Detail of the zinc coordination. The zinc ion is sandwiched between Cys54 and the sulphate group of the MES ion, about 10 Å away from the calcium ion.

While building and refining the protein model, relatively strong density peaks were observed near the terminal atoms of the side-chains of Cys54 and Asp56 in each of the four protein chains in the asymmetric unit, suggesting the presence of metal ions. X-ray fluorescence spectroscopy is a powerful method to identify trace elements in biological samples [18]. Therefore, we recorded an X-ray fluorescence spectrum from a frozen native CHAP_K_ protein crystal, which revealed significant amounts of zinc and calcium (Figure 3A). Sulphur (from methionine, cysteine residues and buffer molecules) and chlorine (from the crystallization buffer) were also detected. The presence of trace amounts of titanium and copper is likely the result of interaction of the beam with certain beamline or sample holder components not related to the sample.

The calcium ion is bound in the amino-terminal part of the protein, involving residues of the long loop connecting the first and second alpha-helices (residues 17–54) and Asp56 in the second alpha-helix. It is bound in a monodentate way to the side chain of residues Asp45 and Asp47 and in a bidentate way to both oxygen atoms of the Asp56 side chain (Figure 3B). Additional ligands are the main chain oxygen atoms of Tyr49 and His51 and an ordered water molecule. The coordination is octahedral and almost exclusively involves carbonyl oxygen atoms, as expected for calcium. Experimentally determined metal ion-oxygen distances are 2.3-2.5 Å, which is also consistent with usual calcium(II) coordination [19]. The occupancy of the calcium site appears to be complete and the refined temperature factors of the calcium ions are very near those of the coordinating atoms (the temperature factors for the calcium ions vary between 10 and 12 Å^2^, while those for the coordinating ligand atoms are between 7 and 14 Å^2^). The calcium ion is near the proposed catalytic site (Figure 2). We propose that the calcium ion plays a structural role, helping to maintain the structure of the amino-terminal domain and thus its catalytic residues in the correct relative orientation. The calcium ion binding loop also contains residues that may be in contact with the substrate and thus play a role in determining substrate specificity. In the derivative protein structure, the calcium is present at the same occupancy and with the same coordinating ligands.

In contrast to the tightly bound calcium ion, the zinc ions appear to be bound more loosely and the derivative structure shows they could be replaced by methylmercury ions upon soaking of the crystals with methylmercury chloride. Also, the occupancy appears to be less than unity, we estimate it to be around 0.67 based on refinement runs performed at different occupancies. Finally, the resulting electron density around the zinc ions is somewhat ambiguous and we could not model the ligands without some remaining uncertainty. The zinc ions are coordinated by the sulphydryl group of Cys54, the sulphate group of the bound MES and several water molecules (Figure 3C). It is also near the main chain oxygen atom of Gly116. The coordination distances for the zinc ion are not ideal; the zinc ion is too close to Cys54 and too far from the coordinating oxygen atoms. A report by another group showed that zinc ions inhibit the LysK enzyme, while calcium ions have no effect on activity, but significantly enhance stability of the enzyme [20]. However, in this assay, metal ions were not removed from the protein solution prior to testing their effects on the enzyme. Zinc ions may play a regulatory role, and their binding near Cys54 suggests they may regulate access of the substrate to the catalytic site.

**Figure 4:**
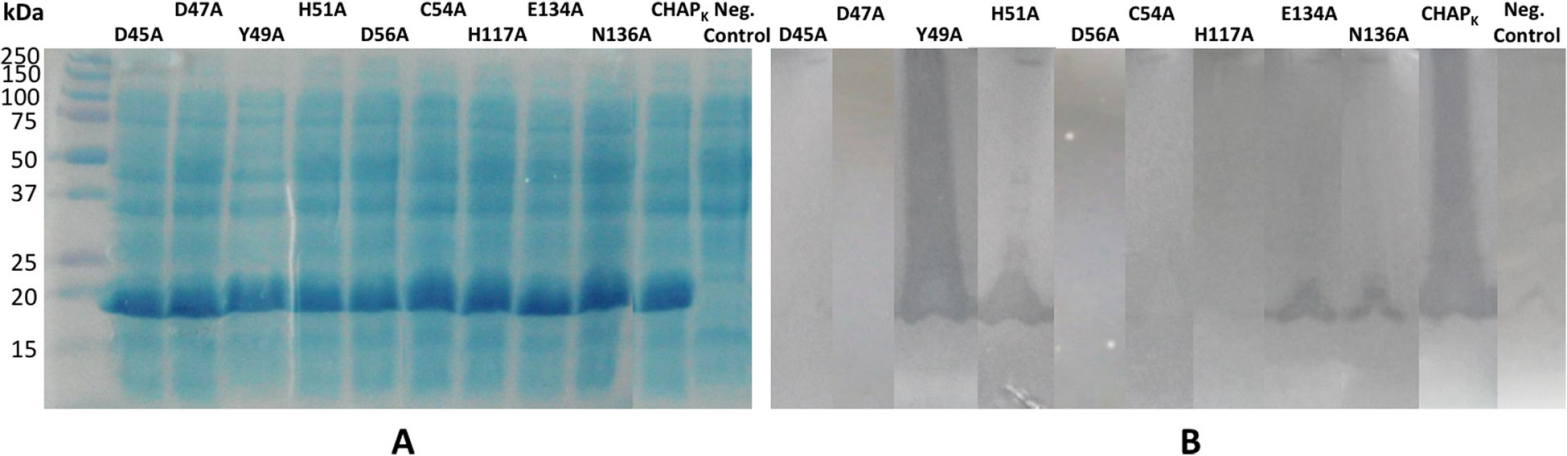
**Overexpression and activity of CHAP**_**K **_**mutants. A**. Sodium dodecyl sulphate polyacryalamide electrophoresis gel of lysates containing over-expressed CHAP_K_ and site-directed mutants. A control not expressing CHAP_K_ is also included. **B**. Composite zymogram gel of CHAP_K_, site-directed mutant CHAP_K_ variants and negative control expression lysates.

The importance of the calcium ion in relation to the catalytic ability of CHAP_K_ was investigated by creation of mutants containing a single amino acid change to alanine at each of the five residues involved in calcium coordination. Zymogram analysis demonstrated that mutation of residues Asp45, Asp47 and Asp56 resulted in the complete abolishment of the staphylolytic activity of the enzyme (Figure 4). This result indicates that the coordinated calcium ion is essential for the catalytic mechanism of the enzyme and complements a previous study, which showed that the chelator EDTA was able to reduce CHAP_K_ activity by 99% [21]. While mutant His51-Ala retained staphylolytic ability, activity of the enzyme was visibly reduced in comparison with the parental CHAP_K_. Mutation of Tyr49 to alanine did not appear to affect the staphylolytic ability of the enzyme as the clearing produced on a zymogram gel was comparable to that seen for non-mutated CHAP_K_ (Figure 4). The fact that mutants His51-Ala and Tyr49-Ala retained activity while the other mutants did not may be explained by the fact that main chain oxygen atoms are involved in coordination as opposed to the side chain oxygens. Therefore these residues are more amenable to substitution without eliminating catalytic activity.

### Catalytic centre and proposed reaction mechanism

**Figure 5:**
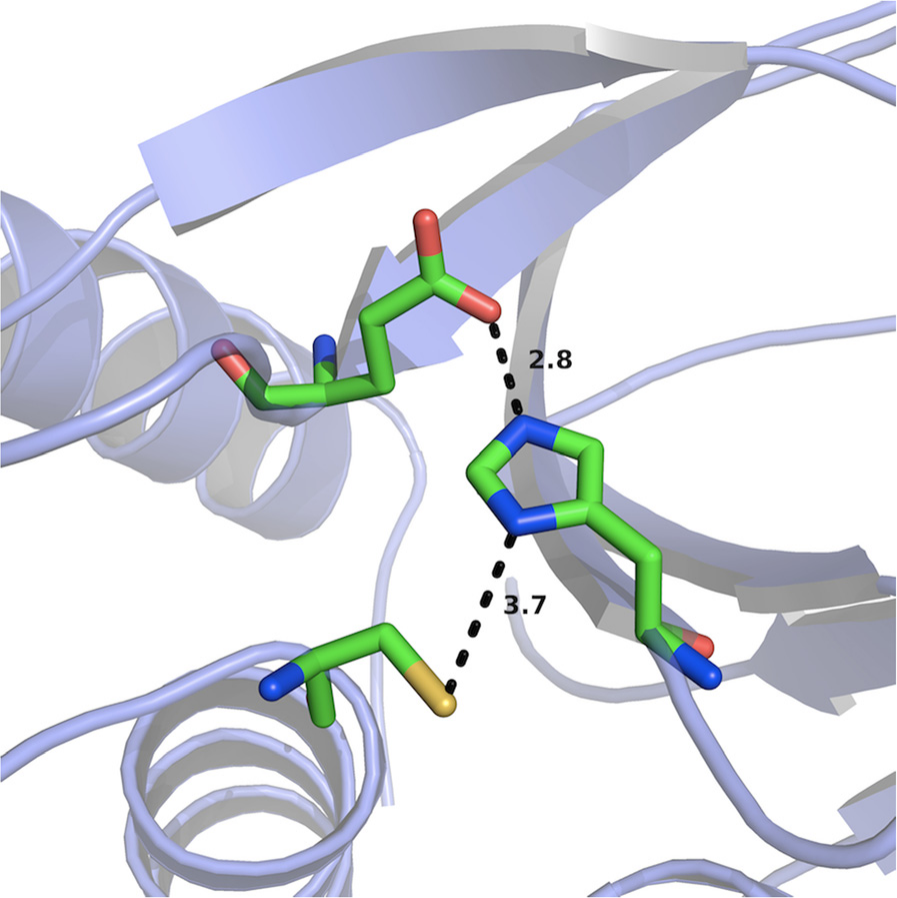
**The proposed catalytic triad of the bacteriophage K endolysin CHAP domain CHAP**_**K**_**.** Cys54 (bottom), His117 (middle) and Glu134 (top) and the distances between them (in Å) are shown.

By comparing the CHAP_K_ protein with other proteins with a similar function and structure (endolysins, CHAP domains and others) and by doing an alignment between them, we can deduce that the catalytic residues are highly conserved. In the CHAP domain of *Staphylococcus saprophyticus* (PDB code 2K3A), the authors describe the presence of a proteolytic triad formed by Cys57, His109 and Glu126 [17], a catalytic triad also found in other members of the CA clan. In the streptococcal phage lysin PlyC (PDB code 4 F88), the catalytic residues are Cys333 and His420 [15], while in NlpC/P60 domain of lipoprotein SPR from *E. coli* (PDB code 3H41) the catalytic residues are Cys68, His119 and His339 [22]. In CHAP_K_ these residues correspond in the alignment to Cys54 located in the second alpha-helix, His117 in beta-strand C and Glu134 in beta-strand D, making these amino acids good candidates to form the catalytic triad of the enzyme (Figure 5). These hypothetical catalytic residues are close to the hydrophobic cleft, which supports the possibility that the catalytic part of the molecule is located in the hydrophobic groove. The predicted pKa of His117 is 9.3. This value contrasts with those of the rest of histidines in the protein: His51 (pKa 5.4), His91 (pKa 6.8) and His 157 (pKa 5.2). His117 may thus be protonated at physiological pH.

Mutation of the conserved Cys54 and His117 residues to alanine resulted in complete elimination of staphylolytic activity of the enzyme as demonstrated by zymographic analysis, indicating an essential role of these residues and supporting the hypothesis that they are part of the catalytic triad. Glu134 is believed to be the other residue of the catalytic triad, but is not as highly conserved as the other two residues. When this residue was mutated to alanine, it was clear from zymogram results that, although the catalytic activity was not completely eliminated, it was strongly reduced. In the absence of Glu134 perhaps another residue can take over its role.

A likely mechanism of action, analogous to that of other papain proteases [23,24], is the following: Glu134 accepts a proton from the protonated imidazole group of His117. His117 subsequently accepts a proton from the hydroxyl group of Cys54 (through its N-epsilon). The deprotonated Cys54 then performs a nucleophilic attack on the peptidic bond between D-Ala and Gly in the staphylococcal peptidoglycan. As a result, a transacylation reaction between the enzyme and substrate occurs, giving rise to an acyl-enzyme intermediate. This intermediate may be hydrolyzed to release the enzyme and the cleaved peptidoglycan [25]. In the NlpC/P60 domain of lipoprotein SPR from *E. coli*, there is a tyrosine residue (Tyr56) that has been reported to be very conserved and which may modulate Cys nucleophilicity or help in substrate binding [22]. In the case of CHAP_K_, Tyr140 is located in an equivalent position, but having a different role, since its phenol group is pointing in the opposite direction. Cysteine proteases have an oxyanion hole, which helps to stabilize the developing negative charge during the formation of the acylenzyme intermediate [26]. Asn136, which is located in close proximity to the catalytic triad, is one residue hypothesized to be involved in creating the oxyanion hole. When this residue was mutated to an alanine, the activity of the enzyme was visibly reduced, but not completely eliminated, supporting the aforementioned hypothesis.

### Comparison with LysGH15 CHAP domain structure

While this manuscript was under review, a paper describing the structures of the CHAP domain (PDB entry 4 OLK), amidase-2 domain (PDB entry 4OLS) and the SH3 domain (PDB entry 2MK5) of the endolysin LysGH15 from phage GH15 was published [27]. The first two were solved by X-ray crystallography at 2.7 and 2.2 Å resolution respectively, while the latter was solved by NMR spectroscopy. Phages GH15 and K share 97% identity in 84% of their genomes (Genbank entries NC_019448 and NC_005880, respectively) [2,28]. The LysGH15 and LysK protein sequences are virtually identical, with only four amino acid differences in their 495-residue sequences. Of the differences, two are in the CHAP domain: Val26 of CHAP_K_ is an isoleucine in CHAP_GH15_ and Glu113 of CHAP_K_ is a glutamine in CHAP_GH15_. The high sequence similarity means the enzymes are almost identical and expected to share the same properties.

When the crystal structures of the CHAP domains are compared, it is notable the spacegroups and crystal packing are very different, which suggests the protein is a monomer in solution and inter-monomer interactions in the crystal are not likely to be biologically relevant. Given the almost identical sequences, it is not surprising that the monomer structures are highly similar; superposition of the two CHAP domains leads to an r.m.s.d. of 0.3 Å when 139 C-alpha atoms are superposed. The only significant difference in main-chain conformation is present in residues 109–116, which follow a different path in the two structures. This may indicate that this loop, which is directed away from the active site, is flexible and of limited importance to the structure and activity of the enzyme. The large side-chains of Tyr49, Trp73, Tyr140 and Tyr153, which are all on the surface of the protein, show different orientations.

The higher resolution of the CHAP_K_ structure when compared to the CHAP_GH15_ structure (1.8 vs. 2.7 Å) should have led to more accurate placement of side-chain atoms and solvent molecules. In both structures, a buffer molecule occupies the groove that likely accommodates the peptidoglycan substrate: a Bis-Tris molecule (2-[Bis(2-hydroxyethyl)amino]-2-(hydroxymethyl)-1,3-propanediol) in between the two monomers of the asymmetric unit of CHAP_GH15_ and a MES and HEPES molecule in the case of the native and derivative structures of CHAP_K_, respectively. The calcium ion is in exactly the same position, as are its coordinating residues and the EF-hand-like domain in which it is incorporated. No zinc ion was observed in the CHAP_GH15_ crystals.

Gu et al. also performed site-directed mutagenesis studies [27], but on the intact LysGH15 enzyme, not on the isolated CHAP_GH15_ domain. As observed for CHAP_K_, it was found that mutating the active site residue Cys54 affected bacterial lysis activity strongly. Mutating the calcium ion coordinating residues Asp45, Asp46 and Asp56 also diminished activity about ten-fold, while Tyr49 and His51 seem less important, the same as we observed.

## Conclusions

We determined the structure of the CHAP_K_ domain of LysK at 1.8 Å resolution (1 Å = 0.1 nm). The structure has the papain-type fold with a long loop between the two amino-terminal alpha-helices. The structure suggests the location of the active site near a hydrophobic groove, with Cys54, His117 and Glu134 forming the catalytic triad. The substrate most likely binds to the hydrophobic groove.

A calcium ion was found tightly bound to the protein. Its ligands are the side-chains of Asp45, Asp47 and Asp56, plus the backbone oxygens of Tyr49 and His51, all in the amino-terminal domain specific to CHAP_K_. It likely has a structural role, stabilizing the protein fold. It may also be involved in ensuring the correct location of the peptidoglycan inside the catalytic cleft or in the stabilization of the negative charge of the tetrahedral intermediate during catalysis. A zinc ion was also found and is likely more loosely bound, as it is less buried, has less protein ligands and could be exchanged for a methylmercury ion upon derivatization. Its role, if any, may be regulatory.

Based on the structure, we propose a possible reaction mechanism, involving all three residues of the likely catalytic triad. Future studies will include co-crystallization with peptidoglycan analogues and elucidating the role of the CHAP_K_ domain in the complete LysK protein. This may allow site-directed mutation to modulate the peptidoglycan specificity and activity of both the CHAP_K_ and LysK enzymes.

## Methods

CHAP_K_ was expressed, purified, crystallized and crystallographic data was collected as described [9,12]. A complete native dataset was collected to 1.8 Å resolution with good statistics. A dataset to 1.7 Å resolution, but with inferior completeness, was also collected from a methylmercury chloride derivative at the Hg L-I edge [12]. However, this dataset allowed phase determination by single anomalous dispersion (SAD) and automatic model building of four crystallographically independent protein molecules in the P1 unit cell [12] (Table 1) using the ARP-WARP program [29]. The model was refined against the derivative dataset and separately against the native dataset. The models were completed and adjusted using COOT [30] and refined with REFMAC5, using local non-crystallographic symmetry restraints [31] and taking care to select the same reflections for calculation of Rfree [32]. To confirm the presence of zinc and calcium ions in the sample, an X-ray fluorescence emission spectrum was collected on a native protein crystal at ESRF beamline ID23-1 [33]. Validation was performed with MolProbity [34]. Refinement and validation statistics are shown in Table 1.

Crystal contact analysis was done with PISA [35]; other analyses were performed with the CCP4 suite [36]. Structural similarity analysis was performed with DALI [37]; for plotting a protein surface coloured according to amino acid conservation, CONSURF was used [38]. The pKa of selected residues in the protein structure was predicted with PROPKA [39]. The structural models and underlying data files have been submitted to the PDB (accession code 4CSH for the native structure and 4CT3 for the derivative). PYMOL (Schrödinger LLC, Portland OR, USA) was used for making structure figures and TOPDRAW [40] to draw the secondary structure diagram.

CHAP_K_ mutants were created using the QuikChange II Site-Directed Mutagenesis Kit from Agilent (Santa Clara CA, USA) as per the manufacturer’s instructions. Crude cell lysate was analyzed for over-expression using sodium dodecyl sulphate gel electrophoresis and for ability to lyse *Staphylococcus aureus* cells using zymographic gels as described previously [41].

## Competing interests

The authors declare that they have no competing interests.

## Authors’ contributions

RK purified the protein and performed site directed mutagenesis and zymogram activity tests; MSG crystallized the protein. MSG and CGD collected X-ray diffraction and fluorescence data. MSG, CGD and MJvR performed structure solution and refinement. MSG, RK, AC and MJvR analyzed the structure. MSG and MJvR drafted the first version of the manuscript. AC initiated the project, while AC and MJvR supervised it. All authors helped to write and improve the manuscript and approved the final version.

## Supplementary Material

Additional file 1: Table S1 Sequence aligment underlining the colour coding of Figure 1D. Click here for file
