# Supplementary material for: Crystal structure of the lytic CHAPK domain of the endolysin LysK from Staphylococcus aureus bacteriophage K
Source: Virol J. 2014 Jul 26;11:133. doi: 10.1186/1743-422X-11-133 (PMC4126393; doi:10.1186/1743-422X-11-133)
Supplement: Additional file 1: Table S1 — Sequence aligment underlining the colour coding of Figure 1D. [file 1743-422X-11-133-S1.pdf]

ConSurf Color-Coded MSA

CHAPk

UniRef90_U6FYE9. Autolysin. Staphylococcus capitis CR01
UniRef90_D3QFD1. Phage N-acetylmuramoyl-L-alanine amidase. Staphylococcus lugdunensis
UniRef90_UPI0002FAEBEA. Amidase. Staphylococcus capitis
UniRef90_H9A139. Lysin. Staphylococcus phage StB20
UniRef90_B9CV69. SH3 domain protein. Staphylococcus capitis SK14
UniRef90_Q4L3Y5. N-acetylmuramoyl-L-alanine amidase. Staphylococcus haemolyticus
UniRef90_D1GQI6. Phage amidase. Staphylococcus aureus
UniRef90_E8SE62. Autolysin. Staphylococcus pseudintermedius
UniRef90_Q8SDJ9. Amidase. Staphylococcus phage phi13
UniRef90_UPI00035CC3BA. Amidase. Staphylococcus epidermidis
UniRef90_Q4Z8Y9. ORF015. Staphylococcus phage Twort
UniRef90_E8SE60. Bifunctional autolysin Atl. Staphylococcus pseudintermedius
UniRef90_O56785. Cell wall hydrolase Ply187. Staphylococcus phage 187
UniRef90_H9A0Q2. Lysine. Staphylococcus phage StB27
UniRef90_I6T7G5. Amidase. Staphylococcus phage vB_SepiS-phiIPLA5
UniRef90_Q859K9. Amidase. Staphylococcus phage 44AHJD
UniRef90_A8D3S8. Amidase. Staphylococcus phage SAP-2
UniRef90_I7KMZ0. Possible N-acetylmuramoyl-L-alanine amidase.Staphylococcus equorum
UniRef90_K8N672. Uncharacterized protein. Staphylococcus simulans
UniRef90_UPI00036FC933. Hypothetical protein. Staphylococcus intermedius
UniRef90_K0U7Q5.Autolysin. Staphylococcus arlettae CVD059

Input_pdb_ATOM_A	AKTQAEINKRLDAYAKGTVDSPYRVKKATSYDPSFGVMEAGAIDADGYYH
UniRef90_U6FYE9_2_161	-KTQSQINKRLRDYKNGVVDSPYRVKRWTSYDASFGAMEPGAIDVDRSYH
UniRef90_D3QFD1_2_162	-KTQSQINARLDTYKNGTVDSPYRVKTWTSYDPAFSTMEPGCVDVDHFYH
UniRef90_UPI0002FAEBEA_2_162	-KTQSQINARLNAYKNGTVDSPYRVKTWTSYDPAFGTMEPGCIDVDHAYH
UniRef90_H9A139_2_162	-KTQSQINKRLRDYKNGVVDSPYRVKHWTSYDASFGAMEPGCIDKDRAYH
UniRef90_B9CV69_2_162	-KTQSQINKRLRDYKNGVVDSPYRVKRWTSYDASFGAMEPGCIDKDHSFH
UniRef90_Q4L3Y5_3_166	-KTQAEINKRLDAYRKGTVDSPYRVKVWTSYDNRFYPMEPGCIDVDKSFH
UniRef90_D1GQI6_2_161	-KTQKQVAERLRAYVNGTVDSPYRVRTWTHYDDDFGTMEPGCIDVDRSFH
UniRef90_E8SE62_11_138	----------------------------------LRWYVGRYIDFDGYWA
UniRef90_Q8SDJ9_11_138	----------------------------------LRWYQGRYIDFDGWYG
UniRef90_UPI00035CC3BA_1_125	-------------------------------------MEPGCIDKDRAYH
UniRef90_Q4Z8Y9_9_144	--------------------------------SYIKSKVNTGTDFDGLYG
UniRef90_E8SE60_20_150	------------------------------------NLIGSGVDVDGYYG
UniRef90_O56785_20_150	------------------------------------NLIGSGVDVDGYYG
UniRef90_H9A0Q2_224_354	--------------------------------NYMYSLKGKYVDFDGMYG
UniRef90_I6T7G5_224_354	--------------------------------NYMYSLKGKFVDFDNRWA
UniRef90_Q859K9_9_139	--------------------------------EWIYKHEGAGVDFDGAYG
UniRef90_A8D3S8_9_138	--------------------------------DWIYKHEGVGVDFDGAYG
UniRef90_I7KMZ0_224_353	--------------------------------EYMHSLKGLFVDFDNAFA
UniRef90_K8N672_224_351	--------------------------------SYMKGLKGRYIDFDKAWA
UniRef90_UPI00036FC933_26_147	--KGVDVDGYYG
UniRef90_K0U7Q5_4_128	-------------------------------------LKGKYVDFDKKFA

Input_pdb_ATOM_A	AQCQDLITDYVLWLTDNKVRTWGNAKDQIK---QSYGTG--FKIHENKPS
UniRef90_U6FYE9_2_161	AQCADLPTDYILWLTDNKYRAWGNAKDFPN---NKFPKG--WKIVENKPS
UniRef90_D3QFD1_2_162	AQCADLPTDYILWLTDNQYKAWGNAKDFPN---NKFPTG--WKVIENMPE
UniRef90_UPI0002FAEBEA_2_162	AQCADLPIDYILWLTDNQYRAWGNAKDFPN---NKFPTG--WKVIENLPS
UniRef90_H9A139_2_162	AQCMDLAIDYVMWLTDNQTEMWGDAKSSIK---NKFPKG--WKIVENKPS
UniRef90_B9CV69_2_162	AQCMDLAIDYVMWLTDNQTEMWGDAKSSIM---NKFPKG--WKIVENKPS
UniRef90_Q4L3Y5_3_166	AQCADLTIDYILWLTDNEFRIRGNAKDAINPKKNKLPEG--WKIVLNRPS
UniRef90_D1GQI6_2_161	AQCMDLVVDYCLWISDNQFRIRGNAKQAID---NPLPKG--WKIIRNERA
UniRef90_E8SE62_11_138	YQCMDLAVDYIYWLLD--IRMWGNAKDAIN---NDFKNM--ATIYENTPS
UniRef90_Q8SDJ9_11_138	YQCADLAVDYIYWLLE--IRMWGNAKDAIN---NDFKNM--ATVYENTPS
UniRef90_UPI00035CC3BA_1_125	AQCMDLAIDYVMWLTDNQTEMWGDAKSSLM---NKFPKE--WKIVENKPS
UniRef90_Q4Z8Y9_9_144	YQCMDLAVDYIYHVTDGKIRMWGNAKDAIN---NSFGGT--ATVYKNYPA
UniRef90_E8SE60_20_150	RQCWDLP-NYIFNRYWG-FKTPGNARDMAW---YRYPAG--FKIYKNTTN
UniRef90_O56785_20_150	RQCWDLP-NYIFNRYWN-FKTPGNARDMAW---YRYPEG--FKVFRNTSD
UniRef90_H9A0Q2_224_354	EQCMDLAVQYVYHITDGTIRMWGNAKDSIL---NVFPKG--WQIVKNTPS
UniRef90_I6T7G5_224_354	YQCMDLAVDYVYHITNGKVRMWGNAKDSIL---NIFPKG--WKIVKNTPD
UniRef90_Q859K9_9_139	FQCMDLSVAYVYYITDGKVRMWGNAKDAIN---NDFKGL--ATVYKNTPS
UniRef90_A8D3S8_9_138	FQCMDLAVAYIYYITDGKVRMWGNAKDAIN---NDFKGL--ATVYENTSS

```
UniRef90_I7KMZ0_224_353        YQCMDLIVDFADHVTNG-YRIWGNAKDLTW---VALPKG--WKLVENTPD
UniRef90_K8N672_224_351        FQCMDVAVDYVYHVTKGKVRMWGDAKDSIR---NVFPTG--WKVIENTPD
UniRef90_UPI00036FC933_26_147  MQCWDLP-NYILKKYWG-FTTWGNANAMAQ---KSNYRGNDFKIYRNTPS
UniRef90_K0U7Q5_4_128          FQCMDLIVDYADHVTNG-YRIWGNAKDLTW---VALPKG--WKLVENTPD


Input_pdb_ATOM_A               TVPKKGWIAVFTSGSY--EQWGHIGIVY-DG---GNTSTFTILEQNWNGY
UniRef90_U6FYE9_2_161          TIPKKGWIAVFTSGTY--AQYGHIGIVY-NG---GNTTSFQILEQNWNGW
UniRef90_D3QFD1_2_162          TVPQKGWIAVFTSGTY--AQYGHIGIVY-NG---GNTNSFQILEQNWNGY
UniRef90_UPI0002FAEBEA_2_162   TVPQKGWIAVFSSGTY--AQYGHIGLVY-DG---GNTNSFEILEQNWNGY
UniRef90_H9A139_2_162          TIPKKGWIAVYTAGTY--SRYGHIGIVY-NG---GNTNSFQILEQNWNGW
UniRef90_B9CV69_2_162          TIPKKGWIAVFTSGTY--DKYGHIGIVY-NG---GNTTSFQILEQNWNGW
UniRef90_Q4L3Y5_3_166          TVPRKGWIAVFTDGTY--WEYGHIGMVY-DG---GNTSRFQILEQNWNGW
UniRef90_D1GQI6_2_161          TVPKQGWIGVNTS-----TYYGHIWLVD-KG---ATQMTMPVIEQNWNSL
UniRef90_E8SE62_11_138         FVPQVGDVAVFRNGIY--KQYGHIGIVY-NS---GNTNQFLILEQNFDGN
UniRef90_Q8SDJ9_11_138         FVPQIGDVAVFTKGIY--KQYGHIGLVF-NG---GNTNQFLILEQNYDGN
UniRef90_UPI00035CC3BA_1_125   TVPKKGWIAVYTSGIY--SRYGHIGIVY-EG---GNTNSFQILEQNWNGW
UniRef90_Q4Z8Y9_9_144          FRPKYGDVVVWTTGNF--ATYGHIAIVT-NPDPYGDLQYVTVLEQNWNGN
UniRef90_E8SE60_20_150         FVPKPGDMAIWTGGNYNWNTWGHTGIVV-GP---STKDYFYSVDQNWNNA
UniRef90_O56785_20_150         FVPKPGDIAVWTGGNYNWNTWGHTGIVV-GP---STKSYFYSVDQNWNNS
UniRef90_H9A0Q2_224_354        YIPPVGAIGVCTTGIY--QEYGHIYLVWDNS---GGTNTQTVLEQNFDGN
UniRef90_I6T7G5_224_354        YVPPVGAIGVCTYGIY--QRYGHIYLVWDNS---GGTNTQTVLEQNFDGN
UniRef90_Q859K9_9_139          FKPQLGDVAVYTNG----QYGHIQCVL--S---GNLDYYTCLEQNWLGG
UniRef90_A8D3S8_9_138          FKPQLGDVAVYTNS----QYGHIQCVT--S---GNLDYYTCLEQNWLNG
UniRef90_I7KMZ0_224_353        YVPPIGTIAVFTKGIY--KKWGHTGLVWDNS---GGTESFVILEQNYDTL
UniRef90_K8N672_224_351        YIPPVASIAVNAAG----EFGHIGLVWSNE---GGTEWFTIIEQNWDNQ
UniRef90_UPI00036FC933_26_147  FVPLPGDWAVWAGSNP-----GHVSIVV-GP---ASTSYFYSCDQNWYTA
UniRef90_K0U7Q5_4_128          YVPPIASIAVFTKGIY--KKWGHTGLVWDNS---GGTETFVILEQNYDTL


Input_pdb_ATOM_A               ---ANKKPTKRVDNYYGLTHFIEIPVKA
UniRef90_U6FYE9_2_161          ---ANKKPSLRWDNYYGLTHFIVPP---
UniRef90_D3QFD1_2_162          ---ANKKPTLRWDNYYGLTHFIVPPV--
UniRef90_UPI0002FAEBEA_2_162   ---ANKKPTLRWDNYYGLTHFIVPPV--
UniRef90_H9A139_2_162          ---ANKKPSLRWDNYYGLTHFIVPPV--
UniRef90_B9CV69_2_162          ---ANKKPSLRWDNYYGLTHFIVPPV--
UniRef90_Q4L3Y5_3_166          ---ANKKPSLRWDNYYGLTHFIVPPV--
UniRef90_D1GQI6_2_161          ---ANLKPKRRLDYYYGCTHFIVPPISS
UniRef90_E8SE62_11_138         ---ANTPASLRWDNYYGCTHFIRPHYKS
UniRef90_Q8SDJ9_11_138         ---ANTPAKLRWDNYYGCTHFIRPKYKS
UniRef90_UPI00035CC3BA_1_125   ---ANKKPSLRWDNYYGLTHFIVPPV--
UniRef90_Q4Z8Y9_9_144          GIYKTELATIRTHDYTGITHFIRPNF--
UniRef90_E8SE60_20_150         NSYVGSPAAKIKHSYFGVTHFVRPAYKS
UniRef90_O56785_20_150         NSYVGSPAAKIKHSYFGVTHFVRPAYKA
UniRef90_H9A0Q2_224_354        ---HNTPAKLRVDNFYGTTHYIVPSF--
UniRef90_I6T7G5_224_354        ---ADTPAKLRVDNFYGTTHYIVPSF--
UniRef90_Q859K9_9_139          GFDGWEKATIRTHYYDGVTHFIRPKFSG
UniRef90_A8D3S8_9_138          GYDGWEKATIRTHYYDGVTHFIRPKFS-
UniRef90_I7KMZ0_224_353        ---ANSPAKLRTDNFEGLTHFIVPDF--
UniRef90_K8N672_224_351        ---ADTPAKLRKNDYSQITHFIVPDF--
UniRef90_UPI00036FC933_26_147  N-WTGSPAYKIKHNYNGVTHFVRPPYK-
UniRef90_K0U7Q5_4_128          ---ANSPAKLRTDNFEGLTHFIVPDF--
```

1	2	3	4	5	6	7	8	9

Variable Average Conserved

- Insufficient data - the calculation for this site was performed on less than 10% of the sequences.
